# Back home, beyond borders: a bioecological study of returnee public health scholars in a globalized health system

**DOI:** 10.1186/s12992-026-01201-3

**Published:** 2026-02-26

**Authors:** Animesh Ghimire, Mamata Sharma Neupane

**Affiliations:** 1 Sustainable Prosperity Initiative Nepal, Baneshwor-31, Kathmandu, Nepal; 2 School of Nursing and School of Public Health, Chitwan Medical College, Bharatpur-5, Kailashnagar, Chitwan Nepal

**Keywords:** International education, Return migration, Public health workforce, Bioecological model, Nepal, Globalization, Health systems

## Abstract

**Background:**

Cross-border postgraduate education is reshaping the public health workforce, yet little is known about what “return” looks like in aid-reliant health systems where authority, resources, and evidence are co-produced across government, research institutes, and international organizations. In Nepal, internationally trained public health scholars re-enter a mixed institutional landscape shaped by global higher-education markets, donor accountability regimes, and transnational procurement and supply chains. This study examines how Nepali returnees translate overseas training into system-facing work upon returning home, and how the timing of key milestones shapes what becomes actionable.

**Methods:**

Semi-structured interviews were conducted with 12 Nepali public health scholars who returned after postgraduate study abroad (five doctoral graduates; seven master’s graduates) and were working in Nepal at two research institutes and two international non-governmental organizations. Positive deviance purposive sampling targeted returnees with identifiable system impact. Data were analyzed using applied thematic analysis, guided by Bronfenbrenner’s bioecological model (micro-, meso-, exo-, and macrosystems), with the chronosystem examined as a cross-cutting influence.

**Results:**

Four interconnected themes described patterned engagement across ecological levels. (1) **Microsystem—reconstituting role and voice at home**: return decisions were anchored in close relationships and early workplace recognition, with a time-sensitive shift from “credentialed outsider” to trusted colleague. (2) **Mesosystem—boundary navigation**: participants described convening and translating across institutions, with coordination accelerating after early probation and crystallizing around budget cycles, grant windows, and emergencies. (3) **Exosystem—rules**,** resources**,** and distant decision-makers**: procurement timelines, ethics review procedures, donor reporting templates, and customs/banking processes frequently determined the feasibility and tempo of reforms. (4) **Macrosystem—national imaginaries**,** credential politics**,** and moral horizons of work**: participants described credentials as opening initial doors, but sustained legitimacy depended on visible delivery amid shifting political and administrative cycles; some trained in regional Asian hubs emphasized the practical transferability of methods and policy argumentation. Across themes, timing mattered: family events, contract endings, fiscal quarters, monsoon disruptions, and crisis periods re-shaped constraints and opportunities.

**Conclusions:**

In this cohort, returnee contribution was bioecological and time-sensitive: agency was not simply “brought home,” but assembled through relationships, cross-organizational brokerage, arm’s-length governance, and national legitimacy contests, all modulated by temporal milestones. Treating return as a time-sequenced ecology of action—rather than skill transfer—can better inform how governments, funders, and academic institutions design re-entry supports, procurement and reporting architectures, and evidence-use processes so that international learning more reliably translates into public value.

**Clinical trial number:**

Not applicable.

## Introduction

### Global context

Cross-border mobility has become a defining factor in the dynamics of education, employment, and governance within the health sector. The labor markets for health professionals exhibit significant structural imbalances, with high-income countries (HICs) persistently engaging in the international recruitment of health workers [1]. In contrast, low- and middle-income countries (LMICs) grapple with sustained shortages of healthcare personnel and exhibit uneven capacity to integrate and retain these professionals within their systems [2, 3]. Reviews from health services research describe a persistent global shortfall of clinicians and public-health staff and document how outward migration and stalled reintegration compound domestic capacity gaps [4]. Simultaneously, the landscape of international higher education has evolved into a multisited industry where scholarships, tuition-based pathways, and professional networks facilitate the flow of students from LMICs to prominent educational hubs in North America, Europe, Australasia, and, increasingly, Asia [5, 6]. The result is a sizeable cohort of “returning experts” whose agency is forged in one system and tested in another. Seminal studies of foreign medical graduates showed that returning is rarely smooth: professionals who do go back to their country of origin often face readjustment and role ambiguity before their contributions stabilize [7, 8]. These early accounts remain instructive for understanding current migration regimes and their effects on health systems.

Momentum surrounding diaspora engagement has intensified alongside the flows of education and labor. Research into medical diaspora initiatives reveals a broadening array of activities—such as short-term missions, university partnerships, advisory roles, remittances, and selective permanent repatriation—while simultaneously underscoring persistent challenges: fragmented coordination, donor-driven priorities, and a lack of substantial evidence regarding lasting systemic impact [9]. Financial globalization underscores a significant trend: remittances to low- and middle-income countries (LMICs) have reached unprecedented levels [10, 11]. However, their alignment with the public sector’s needs, particularly in the health sector, remains inconsistent. Simultaneously, humanitarian emergencies and pandemics reveal how transnational supply chains, regulatory standards, and intellectual property frameworks impact procurement, manufacturing, and ethical approval processes within national health systems [12, 13]. Consequently, globalization shapes both the opportunities and challenges faced by returning health professionals [14]. Understanding the dynamics of return, therefore, necessitates analytical tools that extend beyond individual attributes to encompass the complex environments in which these actions are undertaken.

### Problem focus

While the global picture suggests rising numbers of internationally trained health professionals who aspire to “give back,” the Nepali setting reveals a different configuration of forces. Nepal’s health system is marked by rapid federalization, a dense landscape of international non-governmental organizations (INGOs), and heavy reliance on external funding for priority programs [15, 16]. Procurement rules, customs regimes, and donor reporting calendars cut across routine public-health work, from vaccine logistics to surveillance upgrades [17, 18]. Universities and research institutes compete for grant income, and professional prestige is mediated by credential politics—where foreign degrees can open doors but do not guarantee influence inside domestic bureaucracies. These globalization-linked frictions are further intensified by Nepal’s governance realities, where access, legitimacy, and institutional traction can be shaped by patronage-based sociality and informal networks—often described locally as *afno manchhe* (“one’s own people”)—alongside expectations of deference, reciprocity, and political alignment [19, 20]. In such a context, seemingly “ordinary” professional actions (e.g., earning the right to brief a ministry counterpart, aligning procurement deadlines, or convening across silos) can be consequential precisely because they require navigating not only formal rules but also informal hierarchies and shifting centres of authority. In parallel, the geography of Nepali students studying abroad has diversified: alongside long-standing routes to the United States (US), the United Kingdom (UK), and Australia, regional pathways to India, China, and Japan have expanded [21, 22]. These regional hubs provide training that is often methodologically comparable and administratively closer to Nepali realities, creating heterogeneous returnee cohorts whose skills, expectations, and networks differ. How such cohorts re-enter, navigate, and help steer Nepal’s health institutions remains under-described in the literature.

Existing studies offer useful but incomplete frames for this task. Health-workforce research documents reasons for return (family obligations, career ceilings abroad, national service) and the ambivalence of reintegration, including both upskilling and deskilling dynamics [23]. Diaspora reviews map forms of engagement and the recurring challenge of sustainability [9]. Seminal studies on Latin American foreign medical graduates highlight the psychosocial and professional strains of readaptation, underscoring that return is a process rather than an event [24]. Yet few studies interrogate the political-economy interfaces that returnees must navigate in LMIC settings where aid architectures, higher-education markets, and transnational supply chains intersect with domestic hierarchies and regulatory calendars. This omission matters in Nepal because “making things move” often depends on converting technical insight into locally legible authority under conditions of political turnover, contested legitimacy, and relationship-mediated access—conditions that can render the significance of everyday brokerage invisible to external observers despite its practical importance for implementation [25, 26]. Likewise, most research privileges linear “skill-transfer” narratives or counts of positions held, leaving unexamined the day-to-day work through which returnees translate evidence, broker relationships, and align timelines across institutions. In Nepal, this omission is consequential: policy windows are time-bound; donations and tenders are rule-bound; and public legitimacy is locally negotiated [27, 28]. A design that can make these multi-layered interactions visible—and the ways they unfold over time—is therefore warranted.

### Knowledge gap, rationale, aim, and research questions

These observations collectively highlight a significant gap in our understanding: a lack of qualitative insight into how returning Nepali public health scholars exercise agency across nested environments—ranging from teams and organizations to regulatory systems and national imaginaries. Moreover, this agency is influenced by globalized structures of education, aid, and supply. A qualitative approach is particularly suited to this inquiry, as it can reveal meanings, relationships, and temporal dynamics that are not captured by mere headcounts of returnees or standard program inventories. Bronfenbrenner’s bioecological model provides the conceptual warrant for this inquiry [29]: development is driven by proximal processes that occur within and between the microsystem (immediate settings), mesosystem (linkages among settings), exosystem (distant structures that shape opportunities), and macrosystem (cultural-political orders), all evolving within the chronosystem (time). Adapting this model to the political economy of global health enables an analysis that transcends individual attributes [30], allowing for an examination of how cross-border higher education markets (including scholarship regimes, fee-paying pathways, and regional study hubs), aid architectures (comprising donor indicators, fiscal calendars, and accountability frameworks), and transnational supply chains (such as import standards, customs, and logistics) shape the practices of returnees in Nepal. Consequently, this study seeks to address the following research questions:

#### **RQ1**

How do returning Nepali public health scholars interpret and enact their professional roles within Nepal’s health system across ecological levels (micro, meso, exo, macro) and over time?

#### **RQ2**

In what ways do globalization-related structures—transnational higher-education markets, donor and procurement architectures, and domestic regulatory regimes—enable or constrain their capacity to translate knowledge into system-facing action after return?

By foregrounding nested systems and temporality, the study contributes to debates in migration, mobility, and health policy research by reframing return as an ecology of action shaped by globalization rather than a simple movement of skilled individuals back to their country of origin. It further strengthens Nepal-specific interpretation by situating returnee action within institutional environments where informal networks and political contingencies can condition whose expertise becomes actionable—thereby clarifying why “small” relational and procedural achievements can carry outsized significance for health-system work. In doing so, it extends classic readaptation insights to a contemporary LMIC setting and offers a transferable analytic vocabulary for other contexts where regional training hubs, aid dependence, and supply-chain globalization are similarly in play. 

## Methods

### Study design and theoretical framework

This qualitative study adopted an interpretivist, contextualist stance, viewing return as a meaning-making process embedded in social relations and institutional structures [31, 32]. An applied thematic analysis (ATA) approach—inductive, with theory-sensitized attention [33]—was used to generate patterned accounts of how internationally educated Nepali public health professionals engaged with domestic systems after their return. The design was exploratory–descriptive [34, 35], with an explicit positive deviance purposive strategy [36, 37]: participants were sampled because they had demonstrable, system-facing contributions. This “positive deviance” case-selection strategy deliberately foregrounds returnees whose system-facing work gained traction, in order to make visible the practices and constraints through which impact becomes possible under real-world scarcity and administrative complexity; in methodological terms, it is therefore a form of “selecting on the outcome” (i.e., on identifiable impact) and should be read as mechanism-seeking rather than prevalence-seeking [38, 39]. Accordingly, the analysis does not claim that observed patterns represent “typical” return experiences in Nepal, nor does it treat host-region differences (Anglosphere vs. Regional Asia) as generalizable effects of training location; instead, these contrasts are presented as within-sample, hypothesis-generating observations.

Bronfenbrenner’s bioecological model provided the sensitizing framework [29]. The model was used as a scaffold for attention rather than a deductive template for theme construction: themes were developed inductively from participants’ narratives and then interpreted through ecological concepts [40]. For this study the microsystem comprised family obligations and immediate work teams; the mesosystem captured inter organizational interfaces (research institutes, INGOs, provincial offices, universities, and the ministry); the exosystem referred to arm’s length structures (procurement, ethics, donor reporting, banking and customs); the macrosystem encompassed policy discourses, donor architectures, credential politics, and national narratives of public service; and the chronosystem foregrounded timing (pre return triggers, first year adjustment, 12–18 month consolidation, fiscal calendars, grant cycles, disaster seasons). The chronosystem was treated not as a background descriptor but as analytically consequential, because many constraints and opportunities (e.g., fiscal-year closings, tender windows, monsoon disruptions, leadership rotations) appeared to structure when returnee initiatives could gain or lose traction. This scaffold guided data generation and aided interpretive coherence without constraining inductive theme development. In addition, analytic attention was directed to internal contrasts and disconfirming accounts (e.g., delayed recognition, stalled reforms, or “listening work” before action), so that the ecological frame clarified—but did not pre-determine—the thematic architecture.

### Setting, sampling, and recruitment

Fieldwork was conducted in Kathmandu Valley in partnership with two research institutes and two international non-governmental organizations (INGOs) that lead policy analysis, program implementation, and capacity building in Nepal’s public health sector. Eligibility criteria were: (i) Nepali nationals who had completed a master’s or doctoral degree abroad in public health after which they returned to Nepal; (ii) employed in a system-facing public-health role (e.g., program design, surveillance, analytics, policy support, quality improvement) at one of the four organizations; (iii) at least six months in post to enable reflection on return; and (iv) willingness to participate in a recorded interview in Nepali and/or English.

Sampling followed a positive-deviance purposive logic—returnees were invited if they had an identifiable impact. “Impact” was predefined and operationalized as evidence that is either documentary or corroborated, showing policy adoption, institutionalization of tools or processes, measurable service improvements, or formalized cross-organizational leadership. Therefore, findings are presented as a pathway map of how system-facing engagement developed across ecological levels and over time; they are not prevalence estimates and do not make causal claims about the effects of international education itself.

Recruitment combined venue-based convenience with measured snowballing. The research team displayed invitation posters at four organizations in prominent staff areas. Posters summarized the study purpose, inclusion criteria, voluntary participation, confidentiality provisions, and included a QR code for self-registration. Seven returnees registered interest through this route; eligibility was confirmed by AG, and all seven completed consent and interviews. To extend variation (degree level, host country, organizational role, time-since-return) and reach staff who might not have seen the posters, a measured snowball coupled with a maximum variation strategy was followed [41, 42]: after the interview, a subset of participants were asked to nominate colleagues who met the criteria and were likely to be comfortable receiving an invitation. This yielded five additional participants. The final sample (*N* = 12) comprised six staff across the two research institutes and six staff across the two INGOs, with five doctoral and seven master’s graduates trained in the United States, Australia, India, Japan, and the United Kingdom. No invitees withdrew after consenting. To enable the planned analysis of within-sample heterogeneity, participant attributes relevant to return and engagement were recorded and used as interpretive lenses during analysis, including age/career stage, gender, degree level, host-region exposure (Anglosphere/Regional Asia), institution type (research institute/INGO), and months/years since return.

### Data collection

Interviews were conducted between March and August 2025, in person at neutral meeting rooms or via encrypted video conference, according to participant preference and duty schedules. A semi-structured guide—informed by literature on return migration (e.g., experiences of upskilling/deskilling, reintegration, diaspora engagement), Nepal’s aid and regulatory context, and the bioecological model—covered: (i) pre-return triggers and timing; (ii) first-year adjustment in teams and households; (iii) cross-organizational brokerage; (iv) navigation of procurement, ethics and donor rules; (v) credential politics and narratives of public service; and (vi) reflections on opportunities/constraints across fiscal and disaster calendars. The guide was drafted by AG and MSN (Table 1). Interviews lasted 55–90 min. With consent, sessions were audio-recorded; immediately after each interview, brief field notes documented contextual details, language switching, salient chronosystem markers, and early analytic memos. Interviews were conducted in Nepali, English, or a mix; code-switching for technical and culturally specific terms was encouraged to maximize precision and naturalness.

**Table 1:** Semi-structured interview guide (open-ended items with probes, ecological anchors)

Q.	Core question (open-ended)	Optional probes (use as needed)	Ecological anchor
1	Please walk through the path from your final year abroad to your current role in Nepal.	Key triggers for return (family event, contract end, visa); decision points; who/what most influenced timing; one moment that made the return feel “settled.”	Chronosystem + Microsystem
2	How did the initial months back shape your role and confidence at work?	Early responsibilities, who welcomed/withheld support, a moment of recognition or dismissal, and how that altered your approach.	Microsystem
3	In your immediate team, how has your way of contributing changed since returning?	Example of proposing a change; how you framed evidence; whose approval mattered; outcome and lesson.	Microsystem
4	Describe a time you connected people across organizations to move a decision forward.	Which actors were in the room? What did you do before/during/after the meeting? What outputs (brief, memo, MOU) were created? What made coordination possible?	Mesosystem
5	Think of a flood, disease surge, or supply shock you worked on. What happened, in what order, and what changed because of the response?	Who escalated; decisions under time pressure; what data counted; what would you repeat or change next time.	Mesosystem + Chronosystem
6	Which procedures outside your control most affected your work?	Procurement steps; ethics review; customs/banking; who the key gatekeepers were; how delays were solved or re-routed.	Exosystem
7	How do funding rules and indicators shape what you can actually do?	Quarterly cycles; audit demands; revising indicators; negotiating exceptions during emergencies; example of a successful amendment.	Exosystem
8	How have your degree and training been interpreted within national spaces?	Early introductions based on university/country; when performance outweighed pedigree; differences you notice between South–South and North-trained profiles.	Macrosystem
9	When advocating policy, what forms of evidence are persuasive, and how does that relate to your sense of public service?	Routine records vs. trials; whose standards prevail (ministry, donors, academia); how you reconcile mismatched expectations.	Macrosystem
10	Over the next 2–3 years, what milestones would signal progress, and what conditions might prompt further study or another move abroad?	Organizational changes that would retain you, learning goals, family considerations, and advice to future returnees.	Chronosystem (cross-cutting)

### Data management, transcription, and translation

Recordings were transferred to an encrypted drive accessible only to the authors. AG produced verbatim transcripts. AG translated Nepali segments into English and cross-checked them by MSN for technical accuracy and idiomatic fidelity; differences were resolved through discussion. Transcripts were de-identified using study IDs (e.g., Participant Number, PhD/Masters—Host Country; Research Institute/INGO), removing the names of persons and organizations while preserving role, sector, and degree/host country descriptors needed for analysis.

### Data analysis

ATA proceeded in five iterative stages [33].

**(1) Familiarizations.** Both authors read each transcript at least twice, reviewing field notes and marking chronosystem cues (e.g., months since return; fiscal quarters; crisis events). Reflexive memos captured early hunches about microsystem voice, mesosystem brokerage, exosystem rules, and macrosystem credential politics. **(2) Codebook generation.** An initial hybrid codebook combined inductive descriptors arising from the first three transcripts with sensitizing codes drawn from the bioecological model (micro/meso/exo/macro/chrono), global-health political economy (aid architectures, donor indicators, supply-chain governance), and return migration literature (recognition, legitimacy, translation). Each code had a definition, inclusion/exclusion rules, and example extracts. **(3) Reliability and calibration.** AG and MSN double-coded four transcripts, which represent approximately 44% of the total dataset. Disagreements were reviewed in consensus meetings that emphasized interpretive convergence rather than statistical agreement. Code definitions and decision rules were refined, producing Codebook v2.0, then v3.1 after a second calibration round. **(4) Theming.** Codes were clustered into sub-categories (e.g., “early permission to brief,” “boundary convening rights,” “grammar of donors,” “credential as initial signal”) and then assembled into candidate themes that traced patterned action at each ecological level while explicitly embedding the chronosystem. Matrices examined similarities/differences by organizational type (research institute vs. INGO), degree level (master’s vs. doctorate), and host-country cluster (Anglophone vs. Asian regional). **(5) Review and finalization.** Candidate themes were reviewed for internal coherence and external distinction against the full data set. Negative cases (e.g., delayed recognition beyond a year; stalled exosystem reforms) were interrogated to sharpen boundaries. Departing from the concept of data saturation, we aimed for analytical sufficiency, which was achieved when additional passes produced minimal conceptual novelty and no changes to the theme architecture within the dataset [43, 44]. A worked example of the analytic chain (meaning unit → code → sub-category → category → theme) is presented in Table 2. No qualitative software was used; manual indexing facilitated close engagement with text in two languages and allowed the authors to maintain a transparent audit trail of codebook versions and decisions.

**Table 2:** Analytic chain from meaning unit to theme

Meaning unit (verbatim excerpt)	Code	Sub-category	Category	Theme
“My father’s ill health was a decisive factor; I packed my bags as soon as I graduated… In the institute, the first few weeks were quiet—people watched to see if I would act like I knew better because I had a foreign PhD[…].” (Senior epidemiologist, PhD—US; Research Institute A, 18 months post-return)	Family obligation triggers return; early “voice” contingent on local trust	Proximal duties; probationary recognition	Role negotiation within immediate work and family settings	**Theme 1 — Microsystem: Reconstituting role and voice at home**
“People joked, ‘So the foreign-degree person has come to fix us.’ My first assignment was visiting a municipal clinic that had run out of pregnancy supplements […].” (Program lead, Master’s—Australia; INGO, 3 years post-return)	Relationship-first problem-problem-solving earns credibility	Small relational wins; identity shift from outsider to teammate	Micro-level confidence-building through everyday collaboration	**Theme 1 — Microsystem: Reconstituting role and voice at home**
“About nine months in, I shifted from just being ‘the data person’ to convening conversations. I set up a one-hour roundtable every two weeks—no slides, a two-page brief in plain language. […] we drafted an MOU that helped cut vaccine stockouts in two districts within a quarter.” (Policy analyst, PhD—Australia; Research Institute B, 20 months post-return)	Permission to convene; plain-language synthesis	Cross-organization facilitation routines	Inter-institutional brokerage (research–provincial–ministry)	**Theme 2 — Mesosystem: boundary navigation**
“My role was to bring the provincial pharmacist, the municipal accountant, and our logistics officer to the same table and then let them own the fixes.” (Program lead, Master’s—India; INGO)	Stakeholder pairing; handover of ownership	Co-diagnosis → joint action → local ownership	Bridging organizational logics to enable action	**Theme 2 — Mesosystem: boundary navigation**
“We were authorized to open a water-borne disease testing facility. The PCR machines arrived on time, but the reagents were delayed […]. The donation was meant to help Nepali patients quickly—government processes should support that.” (Laboratory lead, PhD—Japan; Research Institute A, 11 months post-return)	Customs and import bottlenecks; supplier batching	Procedural friction beyond team control	Arm’s-length rules that slow implementation	**Theme 3 — Exosystem: Rules**,** resources**,** and distant decision-makers**
“Our grant promised ‘uninterrupted antenatal supplements in all facilities.’ After the landslide, that was impossible. We proposed an amendment—target the five districts in stock-out and request government funding for air cargo. Initially hesitant, they agreed when we showed evidence. The right clinics received supplies.” (Program manager, Master’s—Australia; INGO, 2 years post-return)	Indicator renegotiation; evidence-based amendment	Donor grammar realigned to local bottlenecks	Navigating funder rules and government co-financing	**Theme 3 — Exosystem: Rules**,** resources**,** and distant decision-makers**
“A US PhD got me onto a national guideline committee in a month, but the advantage faded […]. By year two I was introduced by my last policy delivered, not by my degree.” (Senior policy adviser, PhD—USA; Research Institute A, 2 years post-return)	From credential capital to performance capital	Public legitimacy through delivery	National-level authority defined by outputs, not pedigree	**Theme 4 — Macrosystem: National imaginaries**,** credential politics**,** moral horizon**
“During the pandemic, donors pushed for a large randomized trial on vaccine effectiveness while districts begged for oxygen, ventilators, and protective equipment. Everyone had a different goal—some more grants, some more funding—while hospitals needed oxygen now.” (Program lead, Master’s—India; INGO, 3 years post-return)	Evidence politics vs. urgent need; value conflicts	Reframing “what counts” as impact in crisis	National narratives of service and evidence hierarchies	**Theme 4 — Macrosystem: National imaginaries**,** credential politics**,** moral horizon**

### Rigor and trustworthiness

Credibility was supported through prolonged engagement over the five-month fieldwork window, dual coding of transcripts, and member reflections: anonymized theme summaries were shared with eight participants who had consented to follow-up; all affirmed accuracy with minor clarifications on organizational labels, which were incorporated. Dependability was enhanced by maintaining dated logs of recruitment decisions, interview adaptations, codebook versions (v1.0–v3.1), and consensus rationales. Confirmability was enhanced through reflexive memoing and peer debriefing with the research team. Transferability is addressed through a rich description of setting, organizational ecology, sampling logic, and participants’ characteristics (see Results, Table 3), enabling assessment of applicability to other LMIC settings with similar aid ecosystems.

### Researcher reflexivity

Both authors are Nepali public health academics with experience working across the research institutes and INGO sectors. AG is a returnee scholar with postgraduate training abroad; MSN is a domestically trained qualitative methodologist with extensive collaboration across donor-funded programs. This complementarity was treated as a resource for analytic reflexivity [45]. Prior assumptions—including expectations of faster advancement for returnees or a priori skepticism about donor indicators—were explicitly memoed and revisited during consensus meetings. Role boundaries were maintained to minimize influence on participants’ employment relationships; interviews were conducted outside the organization’s premises when preferred.

### Ethical considerations

The Nepal Health Research Council (109/2025) granted ethical approval. Participants received an information sheet in Nepali and English and provided written informed consent. Participation was voluntary; individuals could withdraw up to two weeks after transcript confirmation. All personal and institutional identifiers were pseudonymized. Audio files and transcripts were stored on encrypted drives accessible only to AG and MSN. No incentives were offered.

## Results

### Participants’ characteristics

We conducted interviews with 12 Nepali public health professionals who returned to Nepal after completing postgraduate studies abroad and who were employed in system-facing roles across two research institutes and two international non-governmental organizations (INGOs) in Kathmandu Valley (Table 3). The cohort included five doctoral (PhD) graduates and seven master’s graduates, with the latest degrees completed in the United States (*n* = 2), Australia (*n* = 4), India (*n* = 3), Japan (*n* = 2), and the United Kingdom (*n* = 1).

Consistent with the study’s attention to globalization-linked opportunity structures, funding pathways reflected cross-border scholarship and fee-paying routes. US returnees received full-tuition scholarships and stipends. The Australian PhD graduate held a Research Training Program (RTP) scholarship with a stipend [46], and the Australian master’s graduates were self-funded. Japanese PhD and master’s graduates received MEXT scholarships from the Japanese government [47]. Indian degrees were supported through tuition scholarships from the Government of India for Nepali students [48]. The single UK master’s student was self-funded. To reduce deductive disclosure in a small, high-visibility professional field, role titles are reported in broad categories, and organizational names remain anonymized.

**Table 3:** Sociodemographic and professional characteristics (*N* = 12)

Participant ID	Gender	Age (years)	Highest degree	Host country (latest degree)	Host region (latest degree)	Employing institution in Nepal	Role category (generalized)	Time since return
1	Woman	28	Master’s	Australia	Anglosphere	INGO A	Programme lead (service delivery / logistics)	3 years
2	Man	37	PhD	Japan	Regional Asia	Research Institute A	Laboratory lead (testing / surveillance infrastructure)	11 months
3	Woman	35	PhD	Australia	Anglosphere	Research Institute B	Statistician / analyst (evidence synthesis)	19 months
4	Man	29	Master’s	Australia	Anglosphere	INGO A	Programme manager (maternal health / commodities)	2 years
5	Man	42	PhD	United States	Anglosphere	Research Institute B	Senior policy adviser (guidelines / policy processes)	2 years
6	Woman	31	Master’s	India	Regional Asia	INGO B	Programme lead (cold chain / emergency response)	3 years
7	Woman	26	Master’s	Japan	Regional Asia	Research Institute B	Researcher (protocols / applied research)	6 months
8	Man	33	Master’s	India	Regional Asia	INGO A	Programme lead (policy/program interface)	1 year
9	Man	50	PhD	United States	Anglosphere	Research Institute A	Senior epidemiologist (system-facing analytics)	2 years
10	Man	31	Master’s	India	Regional Asia	INGO B	Programme lead (coordination / logistics)	14 months
11	Man	36	Master’s	United Kingdom	Anglosphere	INGO A	Programme manager (donor reporting / implementation)	3 years
12	Man	48	PhD	Australia	Anglosphere	Research Institute B	Policy analyst (inter-organizational convening)	20 months

### Findings

**Fig. 1:**
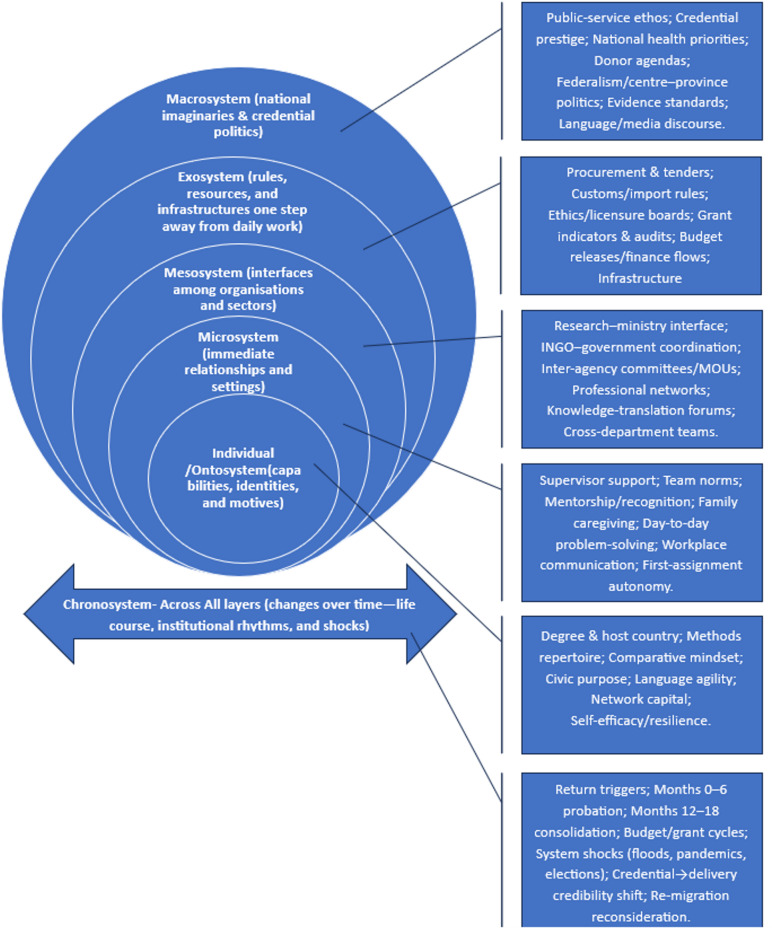
Ecosystem map of returnee nepali public health scholars’ system‑facing engagement (bioecological model)

The analysis identified four nested themes that highlight how returning Nepali public health scholars engage across different ecological levels: (1) Microsystem—redefining their roles and voices within immediate teams; (2) Mesosystem—acting as intermediaries across various organizations; (3) Exosystem—navigating regulations, resources, and distant decision-makers; and (4) Macrosystem—functioning within national narratives and credentialing politics (Fig. 1). While these themes are structured hierarchically (from micro to macro), they are cyclical in practice, as changes in projects, budget cycles, and personal circumstances often shift participants across the ecological levels. We incorporated the chronosystem throughout all four themes, acknowledging that timing significantly impacts engagement. This approach is grounded in participants’ narratives and aligns with established evidence that decisions about whether to return or stay, as well as the readjustment process, typically unfold over 1 to 2 years, influencing future trajectories [49, 50]. To address within-sample heterogeneity, the analysis attends to both convergence and divergence across degree level (Master’s/PhD), institution type (research institute/INGO), and host-region exposure (Anglosphere/Regional Asia), as well as variations related to gender and career stage.

### Theme 1 — Reconstituting role and voice at home *(microsystem)*

In the first few months after returning, immediate relationships—family commitments, supervisors, and work colleagues—shaped why participants came back and how confident they felt about acting. Participants also described a temporal arc: a trigger for return, a probationary period in the first three to six months, and a consolidation phase around 12–18 months when voice and role stabilize. Across accounts, “voice” was not narrated as an entitlement attached to foreign training; rather, it was narrated as a fragile, relational capacity that had to be granted, tested, and re-earned in situ. Notably, “voice” was also described as unevenly available across personal circumstances: in several accounts, the early months involved negotiating credibility while simultaneously managing gendered and family-based expectations about availability, care, and deference—making the probationary period feel like a “double test” rather than a purely professional onboarding.

Across accounts, decisions to return were narrated through proximal ties: an ailing parent, marriage and caregiving, or a wish to raise children near grandparents. These ties interacted with participants’ workplaces, where early recognition—or its absence—amplified or muted their “voice.” As one returnee remarked, “*My father’s ill health was a decisive factor; so I packed my bags as soon as I graduated; I felt I had no choice but to fulfill my obligations at home […].” (Participant 06*,* woman*,* 31*,* Master’s—India; INGO B; 3 years post return)*. While family obligation appeared across genders, women participants more often framed return as time-compressed and non-negotiable because care expectations required physical presence, whereas some men described return as a negotiated settlement between family stability and longer-term career sequencing.

*“When my father got sick*,* everyone assumed I would be the one to manage the hospital visits and the paperwork. At the same time*,* my office treated the first months as my probation. I did not want my team to think I was unreliable*,* so I would do the hospital work early*,* come to the office*,* and then finish reports at night. In those months*,* I learned that ‘voice’ comes after people can point to your consistency—first you show up*,* then you suggest change.” (Participant 06*,* woman*,* 31*,* Master’s—India; INGO B; 3 years post return).*

The tension between familial obligations and workplace demands became most pronounced as participants reflected on their initial months in their roles. Many indicated that if meaningful trust had not been established by the end of the second quarter, they often began to reconsider the options of re-migrating or pursuing further studies abroad. This pattern was especially pronounced in several early-career Master’s graduates, who described the first two quarters as a “make-or-break” period for whether returning could become a long-term trajectory, whereas several mid-career PhD returnees described a longer horizon for consolidation, but also higher initial expectations from colleagues. Accounts also differed by time since return: participants within their first year in Nepal more often described voice as provisional and easily reversible (“one wrong meeting and you are labeled”), whereas those two to three years post-return more often described voice as stabilized through accumulated delivery and repeated trust signals.

*When I told my doctoral supervisor that I was accepting a position in Kathmandu*,* he warned me not to expect the same degree of autonomy. He was right and wrong. At home*,* my wife’s work and our parents’ support mattered*,* which made the decision feel solid. In the institute*,* the first few weeks were quiet—people watched to see if I would act as I knew better because I had a foreign PhD. I learned to ask before I advise. The first time my unit lead said*,* ‘You take the briefing to the Ministry*,*’ it felt like permission to belong*,* not permission to be the loudest voice. (Participant 09*,* man*,* 50*,* PhD—United States; Senior epidemiologist; Research Institute A; 24 months post return).*

Here, family proximity anchors the return, while early micro recognition (being trusted to brief) catalyzes situated confidence. Participants repeatedly framed “voice” as permission coupled with responsibility, not as status imported from abroad. Temporally, this shift generally occurred within a one-year timeframe—aligned with the adjustment period documented in readaptation accounts for returnee professionals [7]. Notably, however, a few accounts suggested that recognition could be delayed when team hierarchies were steep or when returnees were perceived as “too policy-facing” too soon; in these cases, participants described a longer period of deliberate “listening work” before proposing changes. A mid-career woman doctoral returnee described this as needing to “prove seriousness twice”—through output and through availability—before being treated as someone whose voice could shape decisions.

*“I came back with strong technical confidence*,* but in the first months*,* I was cautious about speaking in meetings because I did not yet know the informal hierarchy. At the same time*,* I had family responsibilities that prevented me from staying late in the office. I was concerned that this would be read as a lack of commitment. I compensated by sending analyses early*,* documenting assumptions clearly*,* and volunteering for a high-pressure deliverable to demonstrate my reliability. After that*,* my supervisor stopped introducing me as ‘the returning PhD’ and started saying*,* ‘She leads this workstream.’ That is when my voice became less risky to use.” (Participant 03*,* woman*,* 35*,* PhD—Australia; Statistician/analyst; Research Institute B; 19 months post return).*

Similarly, a Master’s graduate described how “being right” was not enough in the early months: *“I had evidence*,* but I didn’t yet have permission. Until the second quarter*,* my job was to learn who felt threatened and who felt responsible—only then could I suggest a change without it becoming a personal issue.” (Participant 08; man*,* 33*,* Master’s—India; INGO A; 1 year post return).* This “permission before persuasion” narrative appeared across institution types, but was described differently: research-institute returnees more often tied permission to epistemic credibility (methods, writing, uncertainty), while INGO returnees more often tied it to relational reliability (field presence, responsiveness, and being seen to share operational burdens).

Graduates returning from regional Asian hubs (India, Japan) stressed that proximity in training made it easier to calibrate tone in Nepali teams. They arrived with comparable methods and workplace etiquette, easing re-entry and integration into the organizational culture. *“My professors in Tokyo pushed clarity and patience; back here*,* that translates to taking time to explain why we need a protocol*,* not just insisting on one*,*” (Participant 07*,* woman*,* 26*,* Master’s—Japan; Research Institute B; 6 months post return).* Participants added that the first six months functioned like a “probation clock”: small relational wins during this period set the trajectory for the next year.

*People joked*,* ‘So the foreign degree person has come to fix us.’ It stung because my first assignment was very local—visiting a municipal clinic that ran out of pregnancy supplements […]. The real breakthrough didn’t come from a clever strategy; it emerged from simply calling the storekeeper by name*,* inquiring about how inventory moved*,* and then inviting my team to walk the route with me. After that*,* my manager stopped referring to me as “our Australian graduate” and began saying*,* “She’s the one who organized the supply calendar.” This shift also changed the way I introduced myself. (Participant 01*,* woman*,* 28*,* Master’s—Australia; Programme lead; INGO A; 3 years post return).*

This account illustrates the process of identity transformation within the microsystem, highlighting the transition from being a credentialed outsider to becoming a locally recognized teammate through a series of small, relational achievements. Participants chronologically associated these micro-shifts with external time markers—such as budget quarters, grant start-ups, and the workload surges following disasters and pandemics—when their initiatives were most visible and received recognition. Over 12 to 18 months, these moments accumulated into a stable role identity, setting the stage for the boundary work discussed in Theme 2 (Mesosystem). Across women participants in particular, micro-level stabilization was often narrated as contingent on demonstrating both technical competence and sustained availability under competing care demands—an interaction that shaped the tempo of “voice” acquisition rather than its ultimate possibility.

### Theme 2 — Boundary navigation *(mesosystem)*

Returnees described ‘acting’ as connectors across organizations—research institutes, international organizations, provincial health offices, universities, and the national ministry—where practical work of translation and convening transformed analysis into decisions. Importantly, this boundary work followed a temporal arc, gaining momentum after the first three to six months and crystallizing around annual budget cycles, grant calls, and moments of system change stress. Participants frequently underscored that this work was non-trivial in Nepal because institutional mandates are fragmented, responsibilities shift across federal–provincial–municipal levels, and “who can call whom” is often as consequential as what the evidence says.

Participants consistently framed their contributions as efforts to enhance mutual understanding among institutions. They adapted meeting norms, evidence standards, and writing styles to fit various settings while learning the unwritten rules of each organization. As one statistician remarked, *“The ministry communicates numbers differently than a research institute does*,* so I not only translate the figures but also the manner in which we convey uncertainty and risk” (Participant 3*,* woman*,*35*,* PhD—Australia; Research Institute B; 19 months post return).* This connective labor intensified following the probationary period; by mid-year, several participants had gained the authority to convene inter-organizational meetings, marking a significant shift that led to their first formal agreements. In instances where discrepancies emerged, returnees from research institutes predominantly underscored the importance of translation via briefs, modeling, field research, and navigating standards of uncertainty. Conversely, returnees from INGOs tended to prioritize translation through processes such as sequencing, logistical considerations, and the alignment of accountability among stakeholders who typically operate on disparate planning cycles.

*Approximately 9 months following my initial onboarding*,* I transitioned from identifying myself solely as the “data person” to fostering collaborative discussions with stakeholders. I initiated a bi-weekly roundtable—devoid of PowerPoint presentations*,* rather supplemented by a concise two-page brief written in accessible language. During these sessions*,* the provincial officer would propose actionable timelines*,* stating*,* “We can execute this if procurement deadlines are adjusted by two weeks.” In response*,* the ministry representative would confirm*,* “That aligns with our authority.” By the third meeting*,* we successfully drafted a memorandum of understanding (MOU) to formalize the timing adjustments. While the MOU may have appeared modest on paper*,* it significantly contributed to reducing vaccine stockouts in two districts within a single quarter. (Policy analyst at Research Institute B*,* participant 12*,* man*,* 48*,* 20 months post return*,* PhD—Australia).*

In this context, the concept of permission to convene serves as a meso-level counterpart to the notion of “voice” discussed in Theme 1. It represents a marker of organizational trust and grants the authority to facilitate cross-silo collaboration. Several participants described this as “earning the right to call the meeting,” particularly in settings where convening is shaped by seniority, informal networks, or anticipated political sensitivities. *“Some meetings are not about evidence first—they are about relationships first. If the ‘right person’ is not invited*,* the meeting is polite*,* and nothing moves. Once I understood the informal map—who trusts whom*,* who is seen as ‘aphno manchhe’ [one’s own people]—I could plan the sequence: first one-to-one calls*,* then the group meeting*,* then the written agreement.” (Participant 9; man*,* 50*,* PhD—US; Research Institute A; 24 months post return).*

INGO returnees highlighted the importance of practical brokerage, which involves strategically pairing key stakeholders, guiding them through a comprehensive process, and subsequently allowing them to take ownership of the resulting changes. One program lead stated, *“My role was to convene the provincial pharmacist*,* the municipal accountant*,* and our logistics officer*,* facilitating their dialogue and mutual understanding” (Participant 10*,* man*,* 31*,* Master’s—India; INGO B; 14 months post return).* This meso-level coordination became increasingly refined during crises such as pandemics and surges in vector-borne diseases, as well as during post-flood recovery phases. The pressing nature of these situations legitimized and accelerated cross-institutional collaboration.

*During the monsoon flooding*,* we experienced significant failures in the cold chain due to power grid disruptions and generator malfunctions at regional hospitals. Rather than initiating a new project*,* we collaborated closely with the storekeeper and the delivery driver to optimize the logistics route. We recognized that we lacked the technical expertise required to repair the damaged cold chain systems*,* which are being addressed by teams from New Delhi*,* India. In the interim*,* our immediate strategy was to transfer the vaccines to operational cold chain facilities as quickly as possible to maintain their integrity. This necessitated longer travel distances for patients seeking immunization. However*,* in a highly resource-constrained environment*,* such complexities mean there are no straightforward solutions. (Program lead at an INGO B*,* participant 6*,* woman*,* 31*,* 3 years post return*,* Master’s—India).*

This account illustrates the dynamics of mesosystem translation in contexts of scarcity, highlighting processes such as convening stakeholders, co-diagnosing issues, testing interventions, implementing incremental fixes, and rectifying challenges, all while navigating the complexities of emergent problems that lack clear-cut solutions.

### Theme 3 — Rules, resources, and distant decision-makers *(exosystem)*

Participants consistently identified critical bottlenecks and potential breakthrough points in processes adjacent to daily operations, including procurement regulations, donor reporting protocols, ethics review procedures, and banking and customs requirements. They underscored that the timing of interventions played a crucial role in whether innovative ideas gained traction or ultimately faltered. Across accounts, exosystem constraints were narrated as “arm’s length” governance: rules and calendars that were rarely authored by the people implementing public health work, yet decisively shaped what could be delivered and when.

Returnees described how arm’s-length structures—formal procedures they did not control—shaped what could move. Procurement calendars, exchange-control rules, and import duties turned “straightforward” fixes into long games. *“A three-month process extended to nine months once I learned how cold-chain equipment actually enters the country—letters of credit*,* customs*,* testing certificates—each with its own queue*,*”* recalled *a laboratory scientist. (Participant 2*,* man*,* 37*,* PhD—Japan; Research Institute A; 11 months post return).* These frictions were most acute in the first fiscal year after return, when participants were still learning the cadence of tenders and reporting cycles.

*We received an authorization to establish a facility dedicated to testing for water-borne diseases. While the PCR [Polymerase Chain Reaction] machines were delivered on schedule*,* the accompanying reagents experienced a 2-month delay due to the supplier’s decision to wait for a consolidated shipment. Additionally*,* the customs clearance process posed substantial challenges; we were required to produce extraneous documentation*,* which resulted in our consignment being held up in a warehouse for an extended period. An INGO donation supports this project*,* and it is disappointing that the Nepali government has not facilitated more efficient customs clearance. It is critical for the government to recognize that our efforts are aimed at enhancing the health of Nepali citizens and to support us in expediting these processes rather than impeding them. (Laboratory lead at Research Institute A*,* Participant 2*,* man*,* 37*,* PhD—Japan; 11 months post return).*

This vignette illustrates that challenges within the exosystem often stem from procedural inefficiencies rather than technical limitations. Participants associated their learning experiences with a chronosystem framework, emphasizing the importance of navigating bureaucratic processes effectively. They highlighted the need to establish connections with key individuals within the department to mitigate delays and streamline operations. Several participants also described how procurement and customs processes could become unpredictably slow when staff rotated, when signatures were delayed by political or administrative transitions, or when vendors and intermediaries were contested—making timing knowledge (who to approach, and when) a form of practical expertise. *“Sometimes the delay is not ‘technical’—it is political. The file can sit because someone is waiting for a preferred supplier or a preferred signature. In those moments*,* you either give up*,* or you redesign the work to fit the calendar you cannot control.” (Participant 4; man*,* 29*,* Master’s—Australia; INGO A; 2 years post return).*

INGO returnees underscored the grammar of donors—how indicators, audits, and fiscal year-ends bounded action. *“We speak in quarters because budgets do*,*”* said one program manager. *“If the indicator says ‘all clinics’*,* you spend your energy proving coverage*,* not fixing the two clinics where stockouts keep happening.” (Participant 11*,* man*,* 36*,* Master’s—UK; INGO A; 3 years post return)*. Many participants shared how they utilized their training abroad to revise indicators and scopes, ensuring that accountability aligned with actual bottlenecks, particularly during emergencies when exceptions could be made.

*Our grant stated we would ‘ensure uninterrupted antenatal supplements in all facilities.’ After the landslide during the monsoon*,* that wasn’t possible. We proposed an amendment: focus on the five districts experiencing stock-outs that needed urgent attention. We then had to request additional government funding for air transport. They were hesitant at first*,* but we argued that if international agencies are supplying all the goods*,* at least you can chip in to pay the cargo cost. This way*,* the most deserving facilities received the supplements. (Program manager at an INGO A*,* participant 4*,* man*,* 29*,* 2 years post return*,* Master’s—Australia).*

In this context, the exosystem is meticulously negotiated to ensure alignment: a commitment is established, strategies for managing unexpected situations are created, proposals for amendments to uphold that commitment are presented, and a macro-level solution is pursued through engagement with the Nepali government. Participants noted that these renegotiations often occurred when distant decision-makers, such as government officials, initially showed reluctance but became more open to exceptions following lobbying efforts and the presentation of evidence-based arguments.

### Theme 4 — National imaginaries, credential politics, and the moral horizon of work *(macrosystem)*

Returnees transcended the boundaries of teams and organizations as they navigated country-level dynamics—determining what constitutes “real impact,” which credentials denote authority, how donors and ministries define evidence, and the meaning of “service.” They observed these factors evolving over time in response to elections, budget changes, disasters, and the shifting landscape of regional markets. Across accounts, the macrosystem was experienced as a moving horizon of legitimacy: early credibility could be granted through credentials, but it was repeatedly re-tested through performance under Nepal’s shifting political and administrative cycles.

Participants discussed the concept of credential politics as a national language they needed to acquire. Degrees from the US, UK, or Australia provided initial opportunities, while qualifications from India or Japan often facilitated acceptance within professional circles that valued regional familiarity. *“In the first month*,* my introduction hinged on my degree; by the twelfth month*,* colleagues were only interested in whether I could navigate the ministry calendar and deliver results*,*” (Participant 9; man*,* 50*,* PhD—US; Research Institute A; 24 months post return)*. This shift—from symbolic capital to performance capital—was particularly evident among doctoral candidates.

*The recognition of my international qualifications facilitated my rapid inclusion in a national guideline committee within a month. However*,* the initial advantages quickly diminished. Following the first budget hearings*,* a critical question emerged: “Can you adapt our draft to comply with procurement regulations while maintaining scientific integrity?” I discovered that establishing credibility in this context requires public engagement—participating in the entirety of hearings*,* addressing inquiries from various stakeholders*,* and subsequently traveling to provinces to clarify the implications of the guideline for under-resourced clinics with minimal staffing. By the second year*,* my credentials became secondary; I was introduced primarily by the last policy initiative I successfully implemented. (Senior policy adviser at Research Institute B*,* participant 5*,* man*,* 42*,* 2 years post-return*,* PhD—USA).*

The legitimacy conferred at the macro level demonstrated a temporal dimension; the initial advantage gained from possessing a foreign credential diminished over time. This was subsequently supplanted by a demand for observable and sustained performance outcomes. Multiple participants highlighted that the transition in Nepal is particularly pronounced due to the susceptibility of committee access and policy influence to fluctuations in leadership and evolving political agendas. This dynamic suggests that establishing credibility requires continuous engagement and visibility throughout various political cycles, rather than relying on sporadic acknowledgment or recognition.

Returnees also described a moral horizon shaped by national narratives of public service and by debates about what counts as acceptable evidence for policy. Those trained in Asian hubs framed their education as South–South preparation: methods felt transferable, and examples resonated with local realities. *“Studying in New Delhi taught me to argue policy using routine records and what clinic staff can actually do next week with limited resources*,*” a program lead noted* (Participant 8, man, 33, Master’s—India; INGO A; 1 year post return). A parallel issue arose when donors prioritized only randomized trials, while local needs varied.

*During the pandemic*,* the national conversation shifted. Donors focused on conducting a large-scale randomized trial of COVID-19 vaccine effectiveness*,* while districts*,* provinces*,* and hospitals pleaded for ventilators*,* oxygen cylinders*,* and PPE [personal protective equipment]. We know what the priority is*,* but people and organizations are invested in achieving their own goals. Some want more funding*,* others want more grants*,* and some just need oxygen to breathe […]. It’s a crazy world we live in! (Program lead at an INGO B*,* participant 10*,* man*,* 31*,* 14 months post return*,* Master’s—India).*

In this context, the macrosystem undergoes reconfiguration: national stakeholders expand the definition of what constitutes the “right thing to do” in response to crises, while chronosystem disruptions, such as pandemic waves, create interpretative opportunities for this reframing. This highlights the dissonance that can arise when reliance on international donors does not align with local needs.

## Discussion

This qualitative study examined how 12 Nepali public health returnees—trained in the US, Australia, the UK, India, and Japan—re-entered and engaged Nepal’s public health landscape through multilevel, time-sequenced work. Using a bioecological lens with an explicitly temporal (chronosystem) orientation, the discussion synthesizes four themes—microsystem voice, mesosystem boundary navigation, exosystem rules and resources, and macrosystem credential politics—as patterned accounts of system-facing engagement in a purposively selected, positive-deviance cohort. Read together, the themes reposition “return” not as a single event but as an iterative process of repositioning across nested settings within globalized higher education markets, aid architectures, and transnational supply chains that structure opportunity and constraint.

Participants’ first year back was shaped by immediate relationships—with family, supervisors, and colleagues—rather than by credentials alone. Decisions to return were narrated through proximal ties (elder care, marriage, child-rearing) and through an expectation of meaningful work; once in post, confidence cohered around early recognition (“permission to brief the ministry”) and small relational wins that accumulated between months 6 and 18. This temporal arc converges with classic accounts of re-adaptation among foreign medical graduates, which documented that return is episodic and effortful rather than instantaneous, with role realignment unfolding over a year or more [7, 8, 24]. Those early studies emphasized the social and psychological labor of re-entry (e.g., shifting expectations, relearning institutional hierarchies) and remain instructive for understanding contemporary returnee trajectories in the Global South. The present analysis extends these insights by specifying a micro-level mechanism repeatedly emphasized in participants’ narratives: belonging was “earned” through recognition that carried responsibility, not simply granted through possession of a foreign credential. It also nuances review syntheses by Efendi et al. [23], which catalogue reasons for return (family obligations, nationalism, career prospects) and obstacles (deskilling, weak absorption), by showing how proximal ties and early supervisory trust *co-produce* voice and identity in the first quarter back. Within-cohort differences further complicate any singular “returnee experience.” Several early-career master’s graduates described the first two quarters as a high-stakes “make-or-break” interval that could trigger renewed plans for further study abroad if trust was delayed, whereas several mid-career doctoral graduates described a longer consolidation horizon but also higher initial expectations from colleagues and greater scrutiny of tone.

Gendered dynamics were most cautiously interpreted at the microsystem level. In this cohort, caregiving and intergenerational obligations were discussed as key return triggers across participants, and several women participants additionally described how these responsibilities shaped the tempo of the probationary months and the timing of when they felt able to “speak up” at work. Given the small number of women in the sample (*n* = 4) and the positive-deviance design, we treat these observations as suggestive rather than as evidence of a stable gender effect, consistent with wider return-migration research on gendered reintegration and care expectations [51, 52]. On the other hand, any apparent “fit” advantage associated with training in regional Asian hubs is treated here as a within-sample perception: some participants trained in India or Japan described smoother calibration of workplace etiquette upon re-entry, but the small numbers preclude categorical claims about “South–South” versus “North” pathways. Hence, the microsystem theme reframes adjustment as a sequence of situated recognitions through which internationally trained professionals convert symbolic credentials into locally legible relational authority—sometimes quickly, sometimes only after sustained “listening work.”

By months 6–12, many participants shifted from being “the data person” to becoming conveners who translated across research institutes, international NGOs, provincial offices, and the ministry. This boundary work aligns with organizational scholarship on “brokers,” [53, 54] yet the present accounts specify how brokerage is enacted in health policy under globalization: brief, jargon-free synopses; negotiated procurement timelines; and MOUs that stabilize inter-organizational commitments. The pattern complements evidence that diaspora and returnee contributions frequently take the form of short-term missions and partnership-building [55], but it sharpens the mechanism by showing that permission to convene serves as a meso-level analogue to microsystem voice. The study by Frehywot et al. [56] catalogued 89 medical diaspora organizations in the US, the UK, Canada, and Australia, and 68 LMIC diaspora offices and noted persistent barriers—finances, weak sustainability, and communication gaps—without specifying how, in practice, actors translate needs into agreements. The present data fills that gap through concrete routines (bi-weekly roundtables, use of layman’s terms during briefings, explicit alignment of provincial and ministerial calendars) that render collaboration legible and repeatable. Crucially, several participants positioned this brokerage as non-trivial in Nepal because authority is fragmented across federal–provincial–municipal levels and because informal relationship architectures can determine whether coordination is merely performative or actually consequential—captured in participants’ own invocation of “aphno manchhe” to describe how legitimacy and access travel through social ties as well as through formal mandates.

The discussion further develops the concept of “knowledge translation,” which is significant in global health implementation studies [57, 58], by identifying translation not only between “evidence” and “practice” but also among institutions that operate with different norms of evidence (such as statistical uncertainty for researchers and administrative feasibility for provincial officers). The cohort also revealed meaningful institutional variation: research-institute returnees more often described brokerage as a writing-and-evidence craft (briefs, models, field work, research, uncertainty communication), whereas INGO returnees more often narrated brokerage as a sequencing-and-accountability craft (who to convene first, which commitments to formalize, and how to align mismatched calendars). Additionally, the process of brokerage is punctuated by various system stressors—such as pandemic surges, monsoon floods, and transitions at the end of the fiscal year—that heighten the demand for coordination across silos. This chronosystem logic explains why the same professional can oscillate between micro and meso roles over the course of a year: when crises hit, authority to convene expands; when budgets close, it contracts. In contrast with the sustainability concerns raised in the diaspora literature (e.g., mission trips that fade) [9, 59], these returnees negotiated durable process changes (e.g., revised delivery schedules, routinized stock-review meetings), suggesting that proximity and continuous presence can enable forms of institutionalization that episodic diaspora engagements struggle to achieve, while still remaining bounded by shifting mandates and personnel turnover.

Participants consistently identified breakthrough and bottleneck points in structures related to daily work—such as procurement regulations, donor reporting protocols, ethics reviews, exchange controls, and customs—demonstrating how global and national governance shapes local capacity. These experiences recapitulate the barriers catalogued in diaspora reviews [9]—financial strain, lack of follow-through, communication failures, and fragile infrastructure—but add fine-grained accounts of how these barriers materialize in day-to-day implementation. The narrative of PCR reagents delayed by consolidated shipments and onerous documentation requirements demonstrates how transnational supply chains and national customs regulations co-determine the tempo of health innovation. Rather than interpreting delays as mere dysfunction, the exosystem theme shows the “grammar” of donors and regulators in action: when indicators are framed as “all clinics,” managerial energy drifts toward proving coverage; when framed as “resolve stockouts in five districts,” energy concentrates on actual bottlenecks. This reframing resonates with critiques from implementation science, including: using metrics that don’t align with implementation goals [60, 61]. However, the narratives from returnees illuminate the often-overlooked negotiation processes involved, including drafting amendment letters, aligning with fiscal timelines, and compiling evidence to support exceptions in emergency situations. The chronosystem plays a critical role: fiscal year-end deadlines and tender cycles dictate when a promising idea can advance, while pandemic waves temporarily relax regulations. Given that Nepal’s public health system operates within a complex and heavily dependent foreign-aid framework [16, 62], these time-sensitive global processes effectively define the practical horizon for action. This study contributes to the discussion on “absorptive capacity” [63, 64] by demonstrating that returnees do not simply wait for institutional readiness; instead, they learn the rhythms of governance and leverage them to influence feasibility—such as aligning procurement waivers with donor audit timelines or coordinating ethics approvals to coincide with laboratory commissioning. Importantly, several accounts explicitly problematized the assumption that exosystem delay is purely bureaucratic: participants described moments when files stalled around signatures, supplier preferences, or shifting administrative priorities—suggesting that what appears as “red tape” can also reflect politically mediated discretion in procurement and approvals. While prior research often categorized these adaptations as “workarounds,” these accounts reinterpret them as skilled navigation of exosystem rules, which are themselves shaped by the forces of globalization [65, 66].

At the national level, legitimacy was initially conferred through credential politics—degrees from Australia, the US, or the UK opened doors to committees; degrees from India or Japan facilitated acceptance in professional communities accustomed to regional models. Over the first year, however, the source of legitimacy shifted from where a degree was earned to what had been delivered—“performance capital” displaced “symbolic capital.” This finding aligns with research in higher education on the global hierarchy of credentials [67, 68] but challenges the assumption that Euro-American degrees are dominant unconditionally. In this cohort, “credential advantage” functioned as an entry ticket rather than a durable mandate; continued access depended on demonstrating competence within national policy rhythms (hearings, budget cycles, and provincial consultations). Numerous participants noted that the transition in Nepal is particularly pronounced, driven by leadership changes and fluctuating political agendas, which affect committee engagement and policy influence. This volatility means that maintaining credibility relies on consistent visibility and ongoing recognition across different political cycles, rather than on isolated acknowledgments. The analysis thus enhances recent critiques of the “brain drain/skill transfer” dichotomy by illustrating that global education markets can diversify practices [69, 70], and that contextual proximity can serve as an asset rather than a liability. Additionally, it expands the discourse on evidence hierarchies in global health by emphasizing the conflict between donors’ preference for randomized trials and the urgent needs of governments during crises [71, 72]—such as the demand for personal protective equipment during COVID-19 surges and the need for cold-chain recovery following floods. Previous diaspora reviews have acknowledged this tension at the macro level, highlighting the contrast between immediate humanitarian needs and long-term capacity building [9]. However, the present accounts demonstrate how returnees navigate these tensions in real-time by redefining indicators and encouraging government counterparts to co-finance logistics, even when the necessary commodities are supplied externally. The chronosystem once more shapes our understanding: electoral cycles redefine what is considered impactful; phases of a pandemic broaden the types of evidence deemed acceptable; and the development of a project portfolio over 12 to 24 months transitions professional introductions from referencing “foreign-trained” to “leading on X policy.” This aligns with earlier observations that the reputational value of foreign training diminishes as local performance histories are established [73, 74]. The macrosystem theme also clarifies why seemingly “ordinary” competencies (e.g., writing a brief, convening a meeting, renegotiating an indicator) can become politically significant: they re-route legitimacy from pedigree to delivery in a context where visibility, relationships, and administrative timing can be as decisive as technical correctness.

Taken together, the themes portray return as an ecology of action that is continually re-timed rather than a linear translation of learning into impact. At the microsystem level, immediate responsibilities and early supervisory trust convert international credentials into localized authority. In the mesosystem, permission to convene stabilizes cross-silo work in an institutional landscape where fragmentation and informal networks can otherwise keep coordination symbolic. At the exosystem level, donor metrics, procurement regimes, and customs constraints—shaped by global aid markets and transnational supply chains—set the tempo of what becomes feasible. At the macrosystem level, national narratives and credential hierarchies are repeatedly re-tested against performance across political and fiscal cycles. This study, therefore, contributes to scholarship at the intersection of migration, mobility, and health systems by offering a bounded, empirically grounded account of how globalization is “met” in everyday work after return—through calendars, indicators, procurement queues, and legitimacy tests—rather than treating globalization as a background condition. The paper’s conceptual contribution is not to claim that these pathways are universal, but to make analytically visible how time and nested environments co-produce agency in a setting where global education markets and donor infrastructures are deeply interwoven with domestic governance.

## Limitations and future research

As with all qualitative inquiries, this study is bounded by design choices that shape interpretation. The sample was small and intentionally selected using positive deviance purposive sampling to surface information-rich cases of returnee engagement. This design explicitly selects for participants with *identifiable system-facing impact*—in effect, sampling on observed “impact outcomes”—and therefore the findings should be read as mapping *how* returnee engagement can unfold under conditions of relative traction, rather than as a description of typical return trajectories or a basis for prevalence claims. Accordingly, the study is best interpreted as hypothesis-generating, specifying plausible pathways and constraints that can be tested, contrasted, and extended in larger or comparative designs. This strategy privileges depth over breadth and necessarily omits the experiences of returnees who struggled to re-enter, stalled in their roles, or exited the sector. A related limitation is the absence of an explicit comparison group (e.g., domestically trained peers, non-returnees, or stalled returnees), which constrains the ability to distinguish what is distinctive about internationally educated returnees versus what may reflect broader features of professional practice in Nepal’s public health ecosystem. All participants reported having urban upbringings and were employed in research institutes or international NGOs; therefore, perspectives from district hospitals, provincial health directorates, and strictly government roles are not included. Additionally, the cohort was skewed toward men and positions with research or program mandates, which limits the applicability to clinical leadership or regulatory roles. The recruitment process, which combined convenience posters with moderated snowball sampling, may have introduced selection bias favoring highly networked professionals.

The data is based on retrospective, self-reported interviews conducted within the first five years following their return. While efforts were made to encourage openness and to validate findings with a subset of participants, factors such as recall bias and social desirability cannot be entirely discounted, especially in discussions of interactions with ministries and donors. The analysis used an applied thematic approach anchored in Bronfenbrenner’s bioecological model; although the model was used as a sensitising scaffold rather than a deductive template, it may still foreground certain layers of experience (e.g., nested “systems” and timing) while backgrounding other relational dynamics that alternative approaches—such as institutional ethnography, actor-network theory, or political settlement analysis—could surface more sharply. Finally, the chronosystem was reconstructed from participants’ narratives rather than a prospective panel; temporal claims should therefore be treated as indicative rather than definitive. What remains unknown is not only whether the same ecological patterns hold for less-resourced or less-recognised returnees, but also when and why some return trajectories stall—particularly in public-sector-only pathways, rural/provincial postings, and roles where legitimacy is mediated by different authority structures and political incentives.

Future work should expand the scope and method. A longitudinal, multi-site design that follows cohorts at six, twelve, and twenty-four months post return across public, private, not-for-profit, and government sectors would enable stronger chronosystem inference and clarify when micro-level recognition converts into meso-level convening authority. Comparative samples that include non-returnees, stalled returnees, and domestically trained peers would sharpen attribution and reveal counterfactual pathways. Mixed methods designs that link interviews to administrative traces—procurement ledgers, customs clearance times, ethics review queues, donor disbursement calendars—could quantify exosystem frictions and test whether observed bottlenecks predict program delays. Social network analysis and process tracing of specific policy episodes would make brokerage roles and influence channels empirically visible. Cross-country studies across South Asia and other sending regions could examine how regional higher education markets and aid architectures condition credential politics and legitimacy over time. Together, these extensions would strengthen external validity, specify mechanisms operating at each ecological layer, and build evidence to inform policy instruments that support returnee contributions under real-world constraints.

## Conclusion

The study provides an empirically grounded map of the kinds of system-facing work that become possible when returnees secure (i) relational legitimacy within immediate teams, (ii) convening authority across organizational boundaries, and (iii) room to negotiate donor, procurement, and supply-chain calendars that are partly shaped outside Nepal. Conceptually, the study contributes to globalization and health scholarship by making the political economy of return legible within a bioecological frame. It clarifies how cross-border education markets and aid governance are not merely “context,” but enter everyday public health work as credential signals, indicator templates, customs and procurement queues, and the timing of fiscal and crisis cycles that condition what can be delivered and when. The findings also contribute to ongoing discussions on decolonizing global health by showing—at the level of practice—how epistemic authority is contested and how “what counts” as credible evidence for action is renegotiated under shifting conditions of urgency, accountability, and dependence. Read as illustrative, hypothesis-generating pathways for comparable aid-dependent systems, these insights suggest that enabling returnee contributions requires attention to the institutional conditions of trust, coordination, and time—not only to the movement of people. Ultimately, the crucial question for stakeholders is whether global and national actors are willing to redesign the rules and timelines that govern evidence, procurement, and accountability so return can translate into sustained public value.

## Data Availability

The data supporting this study’s findings are available on request from the corresponding author. However, the data is not publicly available due to privacy or ethical restrictions.
